# A role for the cell-wall protein silacidin in cell size of the diatom *Thalassiosira pseudonana*

**DOI:** 10.1038/ismej.2017.100

**Published:** 2017-07-21

**Authors:** Amy R Kirkham, Patrick Richthammer, Katrin Schmidt, Martin Wustmann, Yoshiaki Maeda, René Hedrich, Eike Brunner, Tsuyoshi Tanaka, Karl-Heinz van Pée, Angela Falciatore, Thomas Mock

**Affiliations:** 1 School of Environmental Sciences, University of East Anglia, Norwich Research Park, Norwich, UK; 2 Allgemeine Biochemie, TU Dresden, Dresden, Germany; 3 Tokyo University of Agriculture and Technology, Tokyo, Japan; 4 Sorbonne Universités, UPMC, Institut de Biologie Paris-Seine, CNRS, Laboratoire de Biologie Computationnelle et Quantitative, Paris, France

## Abstract

Diatoms contribute 20% of global primary production and form the basis of many marine food webs. Although their species diversity correlates with broad diversity in cell size, there is also an intraspecific cell-size plasticity owing to sexual reproduction and varying environmental conditions. However, despite the ecological significance of the diatom cell size for food-web structure and global biogeochemical cycles, our knowledge about genes underpinning the size of diatom cells remains elusive. Here, a combination of reverse genetics, experimental evolution and comparative RNA-sequencing analyses enabled us to identify a previously unknown genetic control of cell size in the diatom *Thalassiosira pseudonana.* In particular, the targeted deregulation of the expression of the cell-wall protein silacidin caused a significant increase in valve diameter. Remarkably, the natural downregulation of the silacidin gene transcript due to experimental evolution under low temperature also correlated with cell-size increase. Our data give first evidence for a genetically controlled regulation of cell size in *T. pseudonana* and possibly other centric diatoms as they also encode the silacidin gene in their genomes.

## Introduction

As one of the most successful phytoplankton groups, diatoms contribute ~45% of marine primary production, or 20% of global primary production (Field *et al.*, 1998), and form the base of complex food webs (Smetacek, 1999). Their most characteristic feature is a nanopatterned silica shell (frustule), comprising a hypotheca and overlapping epitheca, which are well conserved in sediments and therefore can be used to reconstruct diatom evolution and climate change based on appropriate proxies. The diatom fossil record shows that frustules have persisted throughout evolution over at least 185 million years (Rothpletz, 1896). It may be assumed that they perform important roles to convey the evolutionary success of the group, given their intricate structure and multifaceted proposed function (including anti grazing defence (Hamm *et al.*, 2003), light modification (De Stefano *et al.*, 2007; Yamanaka *et al.*, 2008; Ingalls *et al.*, 2010) and pH buffering as part of a carbon concentrating mechanism (Milligan and Morel, 2002). Frustule morphology has been used as a tool to identify diatom species since the early 19th century (Mann, 1999). Diatoms are the most diverse algal group, and their species diversity correlates with broad diversity in cell size (Von Dassow *et al.*, 2008), which ranges over more than nine orders of magnitude in cell volume (Litchman *et al.*, 2009).

Size influences many aspects of organisms’ physiology and ecology. Larger phytoplankton cells sink faster than smaller cells owing to their higher weight and lower surface area to volume ratios, which decreases their drag in the water column. For this reason, larger diatoms are responsible for exporting more carbon to the deep ocean than smaller diatoms (Smayda, 1970). This lower surface area to volume ratio also means that larger cells take up nutrients less efficiently, however, large diatoms are able to store more nutrients intracellularly in vacuoles (Stolte and Riegman, 1995). These features are thought to underlie selection for cell size in phytoplankton in general and diatoms in particular.

The diatom fossil record shows a relationship between sea temperatures and average cell sizes (Falkowski and Oliver, 2007). Under increasing temperatures, stratification becomes more widespread in the open ocean, driving selection to smaller size to slow sinking rates and increase nutrient uptake efficiency. Increasing temperatures also increases turbulence and associated nutrient input from deep water in coastal environments due to the increased temperature gradient from open ocean to land (Falkowski and Oliver, 2007), driving selection for larger cell sizes (Sciascia *et al.*, 2013). Satellite and field observations have associated anthropogenic climate change with of 0.8–4.3% annual expansion of stratified gyres (Polovina *et al.*, 2008) and increasing wind-driven mixing in various coastal systems (Bakun, 1990). The latter has been associated with increased relative abundances of large diatom species in the North Atlantic (Hinder *et al.*, 2012). It is therefore likely that phytoplankton cell sizes will continue to respond as global temperatures rise.

The mechanisms underpinning the regulation of cell size have been studied in bacteria, yeast and plants but not diatoms. Generally, in eukaryotic cells, cell size affects internal cellular architecture. The volumes of various organelles are proportional to cell size, and the DNA content scales linearly with cell size (Turner *et al.*, 2012). Thus, evolutionary pressures on cell size influence mechanisms to maintain the most appropriate DNA-to-cytoplasm-ratio (Turner *et al.*, 2012). Most genes involved in cell-size regulation have been identified to be controlling mitosis and translation, and some were found to be involved in loosening the rigidity of cell walls (Cosgrove, 2000). For instance, in yeast, the Wee1 kinase is known to delay mitosis until sufficient growth has occurred. Wee1 mutants enter mitosis before sufficient growth has occurred, leading to abnormally small cells (for example, Nurse, 1975; Marshall *et al.*, 2012). Furthermore, the rate of protein synthesis in yeast seems to be positively correlated with cell size. Deletions of ribosomal genes for instance decreased the average cell size (Soifer and Barkai, 2014). Furthermore, poor growth conditions in yeast and bacteria tend to reduce cell size (Yao *et al.*, 2012). In plants, the loosening of rigid cell walls by expansins allows both elongation and width expansion of cells (Cosgrove, 2000). In most diatom species, it has long been recognised that cells become smaller over generations as smaller hypotheca fit into successively smaller epitheca according to the McDonald-Pfitzer hypothesis (Macdonald, 1869). These cells then undergo auxosporulation and sexual reproduction to restore their maximum cell size (Crawford, 1974). Within species-specific constraints, there is also an environmental modulation of cell size in diatoms induced by temperature, salinity and nutrient supply (Svensson *et al.*, 2014). However, genes and mechanisms underpinning cell-size differences between different diatom species or changes in cell size either according to sexual reproduction or environmental modulation largely remain elusive.

In this work, we have tried to modulate the expression of a frustule component protein termed silacidins. These highly acidic, zwitterionic proteins precipitate silica *in vitro* in the presence of long-chain polyamines and are more concentrated in the biosilica under silicic acid scarcity (Wenzl *et al.*, 2008; Richthammer *et al.*, 2011). Endoproteolytic processing gives rise to three types of silacidins encoded by the same gene (Wenzl *et al.*, 2008). The role of silacidins was therefore considered to be in construction of the frustule by directing longchain polyamines structuring and precipitating silica at low concentration. Surprisingly, we found that the *T. pseudonana* transgenic lines with targeted silacidin deregulation (TSD) resulted in enlarged cells. Comparative RNA sequencing with two of these transformants and the NAT line in addition to previous RNA-sequencing studies for the identification of genes involved in cell-cycle regulation and silicification enabled us to identify a small number of genes potentially also involved in *T. pseudonana* cell size. As the gene encoding the silacidin protein in *T. pseudonana* was also found to be conserved in several different centric diatoms, these data may help to understand processes involved in cell-size plasticity in the group of centric diatoms.

## Materials and methods

### Culture conditions

*T. pseudonana* (clone CCMP 1335) was grown at 20 °C and 24 h light at 100–140 μE, in artificial seawater medium (NEPCC) according to the North East Pacific Culture Collection protocol (http://www3.botany.ubc.ca/cccm/NEPCC/esaw.html). NEPCC medium contains 100 μM concentration of Na_2_SiO_4_. For silica starvation growth experiments, this concentration was reduced to 50 μM and all other nutrients were added at 2 × concentrations, except for vitamin solution that remained at 0.296 μM thiamine, 4.09 nM biotin and 1.48 nM vitamin B12 in all growth media. For nitrate starvation experiments, NaNO_3_ was reduced from 0.55 mM to 0.1 mM, or was completely omitted from the NEPCC with all other nutrients added at 2 × concentrations, except for vitamin solution as above.

Targeted silacidin gene deregulation (TSD) vectors (Supplementary Figure S1) were constructed using standard cloning techniques. A 256 bp fragment of the silacidin gene was amplified from *T. pseudonana* complementary DNA using the primers SILASF (containing *Not*I and *Hin*dIII sites) and SILASR (containing *Not*I and *Eco*RV sites) and inserted into the vectors pTpfcp and pTpNR (Poulsen *et al.*, 2006) using the *Not*I site (additional sites were added to aid in future cloning). The resulting vectors, pTpNRSILAS and pTpFCPSILAS, were sequenced and those containing the silacidin fragment in the antisense orientation were used.

The plasmids were introduced into *T. pseudonana* using the Biolistic PDS-1000/He particle delivery system (BIORAD, Hercules, CA, USA) using M10 tungsten particles according to the method reported by Poulsen *et al.*, 2006. The pTpNRSILAS vector does not contain the antibiotic resistant gene ‘NAT’, conveying resistance to nourseothricin, and thus was cotransformed with the vector pTpFCPNAT (Poulsen *et al.*, 2006; Supplementary Figure 1). Transformed cells were plated onto 50% NEPC medium with 0.8% agar, supplemented with 100 μg ml^−1^ nourseothricin (Werner Bioagent, Jena, Germany). Control cell lines were produced by transforming with the pTpFCPNAT vector only.

### Screening of nourseothricin-resistant TSD transformants

Screening of TSD mutants was initially performed using light microscopy (Olympus BX40) and coulter counter (Beckman multisizer 3 with 100 μm aperture; Fullerton, CA, USA) to observe physical differences between transformant, control (nourseothricin-resistant) and wild-type (WT) cell lines.

The presence of TSD cassettes was confirmed in transformants by PCR using the SILASR primer in combination with either primer pTpFCPt, yielding a 705 bp product, or pTpNRt, yielding an 844 bp product targeting the nitrate reductase or FCP terminator as appropriate.

### Isolation of silacidins

Two different harvesting procedures were used. Cells grown in 20 l silicic acid replete NEPCC medium were harvested during mid-late exponential growth phase by flow-through centrifugation in CEPA High Speed centrifuge Z41 (Carl Padberg Zentrifugenbau GmbH, Lahr/Schwarzwald, Germany). Cultures were harvested from 20 l silicic deplete NEPCC following 48 h silicic acid starvation by filtration onto Isopore 1.2 μm pore-sized membrane filters (Millipore, Billerica, MA, USA). The harvested cells were boiled twice in a lysis buffer containing 0.1 m ethylenediaminetetraacetic acid and 2% sodium dodecyl sulphate. The suspension was centrifuged and washed until the supernatant remained colourless. Diatom silica was dissolved in an acidified ammonium fluoride solution (8 m NH_4_F, 2 m HF, pH 4–5) at room temperature for 25 min. The extract was centrifuged and the supernatant was desalted on a HiTrap column (GE Healthcare, Chicago, IL, USA). The eluate was dried *in vacuo*, dissolved in 250 μl 2 m NaCl and after centrifugation, size fractionated on a Superose 12 10/300 GL column (GE Healthcare; running buffer 200 mM ammonium formate, pH 7.7; flow rate 0.4 ml min^−1^; detection at 226 nm). Fractions eluting between 35 and 38 min (containing silaffin 1/2 l and silacidins) were combined, dried *in vacuo*, dissolved in 2 m NaCl and loaded onto a Superdex-Peptide HR 10/30 column (GE Healthcare; running buffer 10 mM Tris-HCl, 2 m NaCl, pH 7.5; flow rate 0.3 ml min^−1^; detection at 220 nm). Fractions eluting between 33 and 40 min contained silacidins. Recombinant silacidin A’ produced in *Escherichia coli* BL 21 DE3 was used as a standard (Richthammer *et al.*, 2011).

### Transcript level silacidin expression analysis

qRT-PCR was used to establish whether the silacidin gene deregulation was effective at the RNA level as well as at the protein level. 100 ml cultures were concentrated onto Isopore 1.2 μm pore size RTTP filters (Millipore) and flash frozen before RNA extraction with Directzol RNA miniprep Kit (Zymo Research, Irvine, CA, USA). Isolated RNA was treated, complementary DNA synthesized and qRT-PCR performed according to Durkin *et al.* (2009). The primers (SILqPCR-F and SILqPCR-R) were designed to target a region of the silacidin mRNA outside of the antisense fragment encoded by the gene deregulation vectors. Primers used are shown in Supplementary Table S2.

### Imaging and cell measurements

Light microscope images of live cultures were taken using a Zeiss AxioPlan 2ie widefield microscope equipped with an AxioCam HRm CCD camera. For scanning electron microscopy, 15 ml samples of cell cultures were concentrated by centrifugation before treatment with 30% H_2_O_2_, samples were pelleted by centrifugation and washed with deionised water five times before 25 μl resuspended material was mounted onto round glass cover slips mounted on stubs and dried overnight. Stubs were coated in gold particles using a sputter coater and imaged with a Zeiss Supra 55 CP FEG scanning electron microscope (John Innes Centre Bioimaging Facility). For transmission electron microscopy, the diatom cell samples were frozen in liquid propane at −175 °C, then substituted with 2% osmium tetroxide (OsO_4_) in acetone and 2% distilled water at −80 °C for 48 h, before warming to −20 °C for 4 h and 4 °C for 1 h. Samples were then dehydrated twice each in anhydrous acetone and ethanol for 30 min at room temperature. Samples were then continuously dehydrated in ethanol at room temperature overnight before being infiltrated with PO (propylene oxide) twice for 30 min each, and put into a 70:30 mixture of PO and an epoxy resin (Quetol-651; Nisshin EM Co., Tokyo, Japan) for 1 h. Then, PO was volatilized overnight. The samples were transferred to a fresh 100% resin and polymerized at 60 °C for 48 h. The resins were ultra-thin sectioned at 70 nm with a diamond knife using an ultramicrotome (Ultracut UCT; Leica, Vienna, Austria), and mounted on copper grids. They were stained with 2% uranyl acetate at room temperature for 15 min, washed with distilled water, and secondary-stained with lead stain solution (Sigma-Alderich Co., Tokyo, Japan) at room temperature for 3 min. The grids were observed by a transmission electron microscope (JEM-1400Plus: JEOL Ltd., Tokyo, Japan) at an acceleration voltage of 80 kV. Images from light, scanning and tranmission electron microscopy were used to measure cell dimensions and frustule thickness using ImageJ software. Measurements of cell diameter and length from light microscope images of cells in girdle-band orientation were used to calculate surface area and volume, and surface area to volume ratios for individual cells.

### Silicon quantification

Cell samples corresponding to 4 or 6 × 10^8^ cells were collected by centrifugation for each of three replicate samples of WT and TSD cells, respectively. The cells were transferred to wells of an AcroPrep Advance 350 plate (0.2 μm Supor, Pall, Port Washington, NY, USA) and washed four times with MilliQ water before extracting with 100% methanol until residue remained yellowish or colourless. Samples were washed a further four times with MIlliQ water and silica was dissolved at 95 °C for 1 h using 60 μl of a 2 m NaOH solution. Samples were centrifuged (3220 g, 15 min, room temperature) and the flow-through was collected in a new 96-well plate. Another incubation with 20 μl 2 m NaOH solution was conducted to achieve complete silica dissolution and after centrifugation, the flow-through collected in the same plate. The volume of the combined fractions was volumetrically determined using microliter pipette. The amount of dissolved silica in these samples was determined by the molybdenum blue test (Ramachandran and Gupta, 1985). Resulting silica concentrations were divided by cells per sample to give values for silica per cell.

### Growth experiments

Cell lines were grown in batch cultures of 250 ml in triplicate for each growth experiment. Cultures were grown according to culture conditions above. Daily measurements were taken for cell counts (coulter counter, Beckman), photosynthesis based on the quantum yield of photosystem II (F_v_/F_m_; Phyto-PAM-ED, Walz), and light microscopy (Olympus BX40) was used to assess the average number of cells per particle in order to adjust cell counts to allow for cell aggregation. Growth rates were calculated as the slope of the natural logarithm of cell numbers during exponential phase growth.

### Si(OH)_4_ uptake

Samples were taken daily during a growth experiment for analysis, and cells were removed by filtration through isopore 1.2 μm pore size RTTP filters (Millipore), before a second filtration through 0.2 μm pore size Minisart filters (Sigma-Aldrich). Samples were analysed using a Skalar SAN++ continuous flow analyser.

### Aggregation analysis

During the same growth experiment, a sub-sample was taken from each replicate culture and viewed using light microscopy (Olympus BX40). At least 100 cells were counted per sample in triplicate and the number of cells per aggregate was recorded. In addition, the number of cells counted was divided by the number of aggregates counted to give the average number of cells per particle. These data were used to normalise cell abundances obtained by coulter counter for the same cultures.

### Transcriptome sequencing

RNA was extracted from triplicate cultures of nourseothricin cassette alone (NAT) and two independent TSD cell lines harvested during late exponential phase and following 48 h silicon starvation as described under ‘Transcript level silacidin expression analysis’ (Supplementary Figure S2). RNA sequencing was performed according to the Illumina TruSeq RNA protocol by The Earlham Institute (Norwich Research Park). Reads were aligned to the assembled *T. pseudonana* genome using the Tophat program (https://ccb.jhu.edu/software/tophat/index.shtml). Differentially expressed genes between the NAT control and TSD cell lines were retrieved according to twofold (1 × log^2^), *P*<0.01 differential regulation criteria (Supplementary Table S1).

Testing for overrepresented Interpro domains and GO terms was performed using the default Wallenius approximation method using a 0.05 false discovery rate cutoff (Benjamini and Hochberg, 1995). Overrepresented interpro domains are given in Supplementary Table S2. Protein family (pfam domains) assignment was used from the *T. pseudonana* JGI genome website (http://genome.jgi-psf.org/Thaps3/Thaps3.info.html) to designate predicted functions for differentially regulated genes.

### Silacidin and size regulation in *T. pseudonana* under batch cultivation vs experimental evolution

*T. pseudonana* was cultivated semi-continuously at 22 °C and 9 °C, by performing transfers every third day, before cultures reached stationary phase. Prior to this, cultures were maintained in batch culture conditions. Samples were taken from the *T. pseudonana* used to inoculate the experimental cultures before the start of the experiment (T0, batch cultivation), as well as after 300 generations (T300, experimental evolution) at each of two temperature regimes (T0-22 °C; T300-22 °C; T300-9 °C). Light microscope imaging and transcriptome sequencing were performed as described above. Transcript abundances were calculated as reads per kilobase of gene model per million mapped reads. Abundances for the silacidin gene ID 268311, silaffin 1 and 3 (IDs 11 366 and 25 921, respectively), actin-like housekeeping gene (ID 269504, Durkin *et al.*, 2009), and genes associated with silacidin deregulation in TSD1 and 3 under both late exponential and silicon-starved conditions identified by transcriptome sequencing (IDs 23685, 8616, 7349, 23671, 23686, 7435, 9840, 7353, 264048, 263350, 3898, 8776, 12137, 6886, 6681, 7687, 8615 and 9371) were extracted for each sample. Extensive analysis of this experimental evolution study is in preparation for further publication (Schmidt *et al.*, in prep).

### Sequencing of silacidin homologues from centric diatom species

Primers were designed based on the *T. pseudonana* mRNA sequence from RACE-PCR (Richthammer *et al.*, 2011) and targeted the 5’ and 3’ untranslated regions as well as internal exon sequences. PCR was challenging owing to the highly repetitive nature of the silacidin gene sequence. Primers (shown in Supplementary Table S3) were used in each of the four possible combinations. Hot-start, touch-down PCRs in a final volume of 50 μl with 1 mg ml^−1^ bovine serum albumin, were performed with an initial denaturation of 95 °C for 10 min, after which *Taq* polymerase, dNTPs and primers were added, followed by a touch-down phase of either 15 or 45 cycles of 95 °C for 30 s, 65–50 °C for 30 s and 72 °C for 1 min, followed by 15 or 45 cycles of of 95 °C for 30 s, 50 °C for 30 s and 72 °C for 1 min bringing the total number of cycles to 60. A final extension step of 72 °C for 10 min before amplicons were electrophoresed on a 2% agar TBE gel, and the longest amplicons for each species were selected for sequencing. Amplicons were cloned into the PCR2.1 sequencing vector using the Invitrogen original TA cloning kit for sequencing (Eurofins, Luxembourg, Europe). Obtained sequences were translated to amino acid sequence using the ExPASy translate tool (http://web.expasy.org/translate/) and aligned with ClustalW (http://www.ebi.ac.uk/Tools/msa/clustalw2/). Ribosomal 18 S PCRs were performed according to Jahn *et al.* (2014) for all species to confirm that all samples were compatible with PCR.

## Results

Initial observations showed that 79% and 88% of the 22 and 25 nourseothricin-resistant clones obtained for TSD-NR (Nitrate Reductase promoter) and TSD-FCP (Fucoxanthin Chlorophyll Protein promoter) transformations, respectively, had average cell diameter >4.5 μm, whereas WT and clones transformed with the NAT vector only had average cell diameters <4 μm. Direct PCR confirmed that all clones with increased cell size contained the antisense silacidin fragment.

**Figure 1:**
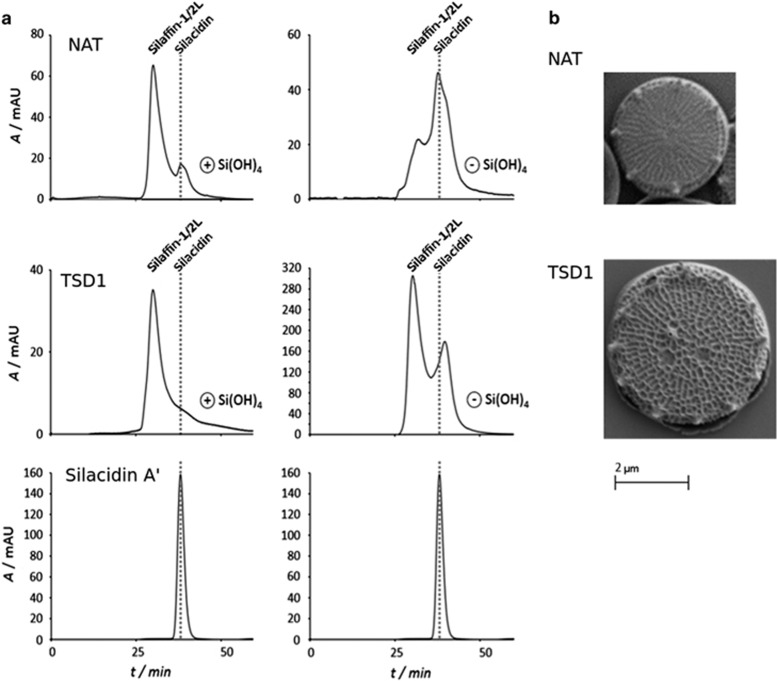
(**a**) Size exclusion chromatograms of low molecular weight proteins isolated from the biosilica of NAT control (top) and TSD cell line in silica replete cultures (left) and silica deplete cultures (right). Silacidin A was used as a standard (bottom). Dotted grey line indicates the elution time of silacidin proteins. (**b**) Scanning electron micrographs of NAT control TSD1 cell line cells harvested from exponentially growing cultures. Scale bar representing 2 μm applies to both images.

Further characterisation was performed on a clone transformed with the TSD-NR vector compared with a NAT clone. Phenotypes described were supported by characterisation of a further two clones transformed with the TSD-FCP vector (see Supplementary Figure S3A and Supplementary Table S4 for details of the vectors). Abundance of the silacidin transcript was not significantly altered in TSD cells compared with NAT or WT, as previously observed in (Shrestha and Hildebrand, 2015). Because a clear reduction of RNA levels was not observed and antibodies to test silacidin protein levels were not available, we performed size exclusion chromatography (Figure 1a). Under exponential growth, silacidins from NAT control cells formed a clear peak, but could not be distinguished in the biosilica from the TSD line (Figure 1a). In biosilica from silicic acid starved cultures, silacidins were more than twice as abundant as silafin-1/2 L and eluted at 33–40 min (as typically observed for *T. pseudonana* silacidins (Wenzl *et al.*, 2008; Richthammer *et al.*, 2011) whereas in the TSD line biosilica silacidins were <60% as abundant as silafin-1/2 L and eluted at 36–43 min possibly indicating degradation or malformation of the proteins.

Scanning electron microscopy, revealed that the TSD frustules had very similar characteristic nanopatterning, but valves were larger and had more central and peripheral pores present per frustule (Figure 1b, Supplementary Figure S3A, Supplementary Table S1). The TSD cells had an average valve diameter of 4.62±0.05 μm compared with 3.7±0.62 μm, 11.44±1.77 fultoportulae compared with 8.6±1.83 and 1.03±0.64 central pores compared with 0.69±0.54 for NAT cells. Transmission electron microscopy (Supplementary Figure S3B) showed no difference in the thickness of cell walls of WT and TSD3 cell lines, with average thicknesses of 48.5±11 nm and 47.9±8.5 nm respectively based on 19–30 individual measurements of each of seven and nine cells for WT and TSD3 lines, respectively. Scanning electron micrographs were used to measure valve diameters (WT, *n*=127; TSD1, *n*=148; TSD2, *n*=19; TSD3, *n*=98), and light microscopy was used to measure frustules in girdle-band view (WT, *n*=50; TSD1, *n*=30; TSD2, *n*=6; TSD3, *n*=9) to calculate average cell volumes and surface areas. This revealed 127.93±30.01 μm^3^ biovolume and 141.85±23.52 μm^2^ surface area corresponding to surface area to volume ratios of 1.128±0.09 for TSD cells compared with 57.79±24.28 μm^3^ biovolume and 82.3±23.61 μm^2^ surface area corresponding to surface area to volume ratio of 1.52±0.23 for NAT cells. Thus, enlarged TSD cells had significantly lower surface area to volume ratios than NAT control cells (*P*<0.01, student’s *t*-test).

Silicon was measured as 159±4 fmol per cell for WT cells and 212±21 fmol per cell for TSD1 cells. When the larger surface area of TSD1 cells is taken into account based on the calculated average cell sizes (Supplementary Table S1), this is actually lower for TSD1 cells than WT cells, at 1.92 fmol μm^−2^ for NAT and 1.5 fmol μm^−2^ for TSD1. However, transmission electron microscopy showed no difference in cell wall thickness between TSD and WT. Thus, it is likely that the higher silica content of TSD cells is partially due to the larger surface area of the cell wall. Our methodology quantifies total cellular silicon, that is, including internal silicon as well as the silica cell wall, and this internal silicon may account for differences between TSD and control cells.

**Figure 2:**
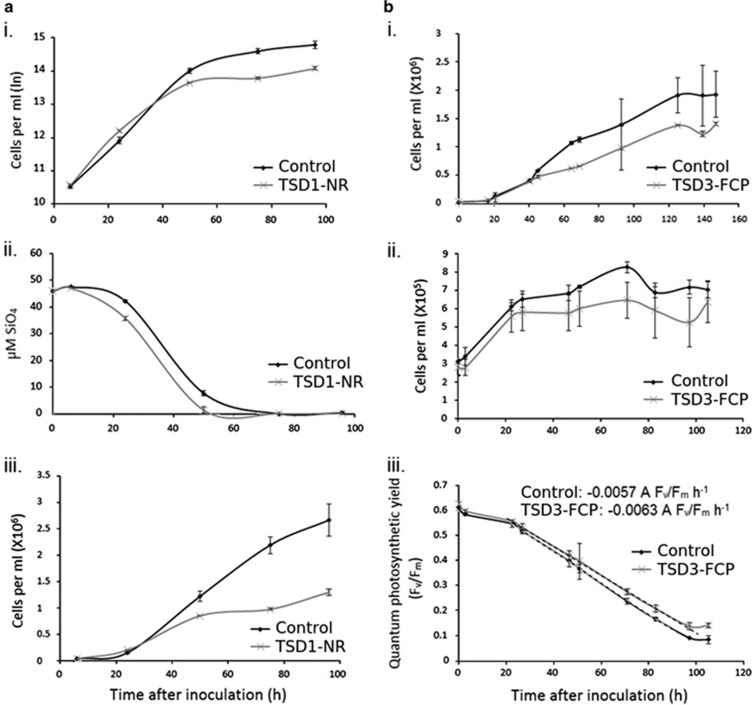
(**a**) Growth of TSD1 - NR cells compared with a NAT control cells in silica-starved medium. i: natural log of cell abundance per ml; ii: Si(OH)_4_ content of the medium; iii: cell abundance per ml. (**b**) Growth of NAT and TSD3-FCP cells under nitrogen starvation i: cell abundance per ml in nitrate-starved medium (containing 0.1 mm NaNO_3_ at the beginning of the experiment); ii: cell abundance per ml after transfer from replete to nitrogen-free medium; iii: quantum photosynthetic yield (F_v_/F_m_) of cultures after transfer from replete to nitrogen-free medium. The slope of change in quantum photosynthetic yield over time is given and highlighted by dashed lines. Error bars represent s.d. of biological triplicate samples in all graphs.

The enlarged TSD cells displayed average growth rates during exponential growth that were not significantly different to NAT or WT cells (1.71±0.42 for NAT cells, compared with 1.79±0.2 for TSD, *P*>0.1 based on average growth rates for three replicates over five different growth experiments). However, many other phenotypic differences were observed. TSD cultures reached lower cell densities (1.3 × 10^6^±3 × 10^4^ cells ml^−1^ for TSD compared with 2.66 × 10^6^±3 × 10^5^ cells ml^−1^ for NAT cells; Figure 2) in silica-starved media, and had increased silica per cell (61.6±2.48 pmoles silica per TSD cell compared with 40.1±5.33 pmoles NAT cell). A different cell line, transformed with the FCP gene deregulation vector similarly showed reduced cell densities in nitrate-starved medium compared with NAT cells (1.4 × 10^6^±2.6 × 10^4^ cells ml^−1^ for TSD compared with 1.93 × 10^6^±4.1 × 10^5^ cells ml^−1^ for NAT cells; *P*<0.01). We also observed that when transferred to N-free medium, exponentially growing TSD cells’ quantum photosynthetic yield fell more slowly than that of NAT cells (slope of 0.0057 Δ F_v_/F_m_ h^−1^ for TSD compared with 0.0063 for NAT; Figure 2). TSD cultures showed lower aggregation than NAT cells during silica starvation (91% of TSD cells were observed to be dispersed as single cells or joined to no more than one other cell compared with only 45% of NAT cells after 48 h silica starvation; Supplementary Figure S4).

**Figure 3:**
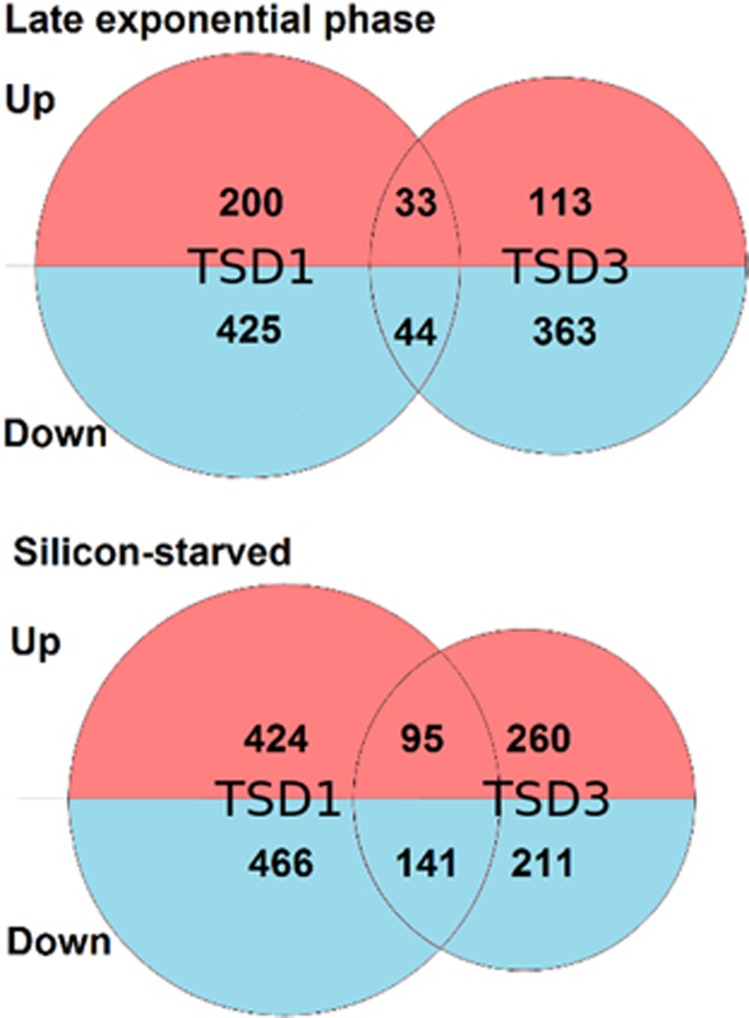
Representation of the numbers of genes found to be significantly differentially regulated in TSD1 and TSD3 cell lines compared with NAT control cells in late exponential (top) and silicon-starved (bottom) conditions. Venn diagrams are to scale with total numbers of differentially regulated genes identified in TSD1 and TSD3 with overlaps corresponding to the number of genes found to be differentially regulated in both TSD cell lines compared with NAT. The numbers of up- and downregulated genes identified are given in the red and blue sections of the diagrams, respectively.

These data suggest that some features of the cell wall (for example, fultoportulae number) scale with cell size. Similarly, we found that RNA content of NAT and TSD cell lines scaled with cell size (*P*>0.01; Supplementary Table S1). We performed transcriptome sequencing of NAT and two different TSD cell lines, TSD1 (TSD1-NR) and TSD3 (TSD3-FCP) harvested during late exponential phase and after 48 h stationary phase caused by Si(OH)_4_ starvation. The expression of over 90% of the genes in the genome are not significantly differently regulated in the TSD cell lines compared with the NAT control line (using the criteria *P*<0.01; fold change>1 log^2^; Figure 3a). Of a total of 11 390 genes in the *T. pseudonana* genome, 233 were upregulated and 469 were downregulated in TSD1-NR, and 146 were upregulated and 409 were downregulated in TSD3-FCP compared with NAT in late exponential phase. In stationary phase, 519 were upregulated and 607 were downregulated in TSD1-NR, and 356 were upregulated and 352 were downregulated in TSD3-FCP compared with NAT (Figure 3). These data were compared with the genes identified in other transcriptomic studies in *T. pseudonana* (Table 1). Silafin*-*like response genes and silicon-starvation responsive genes were identified by Shrestha *et al.*, in 2012; and genes responsive to silicon limitation (Mock_Si), iron limitation (Mock_Fe), nitrogen limitation (Mock_N), temperature limitation (Mock_T) and alkaline pH (Mock_pH) were identified by Mock *et al.* (2008). We selected both studies (Shrestha *et al.*, 2012, Mock *et al.*, 2008) for our comparative approach because Shrestha *et al.* (2012) enabled us to identify genes in our data set with similar regulation patterns to genes encoding well-known cell-wall proteins such as silaffins, and the study by Mock *et al.* (2008) matches the growth condition (silicon limited growth) under which the silacidin protein is most abundant in cell-wall extracts of *T. pseudonana* (Richthammer *et al.*, 2011). The other growth conditions in the study of Mock *et al.* (2008) were used to specifically identify those genes that were only responsive to silicon limitation. We used a similar approach in this study.

Most of the genes (~65%) associated with silacidin deregulation did not feature in either of the two transcriptomic studies. Genes influenced by silicon limitation (Mock_Si) accounted for ~17% of genes influenced by silacidin deregulation in the two mutants and two growth conditions, however, these were not the same genes in each case.

Eighteen genes were found to be differentially regulated between both in TSD lines and the NAT under both late exponential and silicon-starved conditions. Of these eighteen, only seven had any annotation, including ProteinID 264048, which has significant homology to protein kinases and is predicted to contain a proline-rich region. Proline-rich regions are known to be present in diatom cell-wall proteins (Kröger *et al.,* 1997). This gene was also identified as a silifin-like response gene (Shrestha *et al.*, 2012). Two (Protein IDs 8776 and 9371, both with unknown function) were denoted silicon starvation response genes (Shrestha *et al.*, 2012) and an additional three were found to respond to silicon limitation and/or alkaline pH (Mock *et al.*, 2008). The other 12 were not found to be differentially regulated in the other two studies (Mock *et al.*, 2008; Shrestha *et al.*, 2012). The regulation of several genes was confirmed by qRT-PCR (Supplementary Table S3).

**Figure 4:**
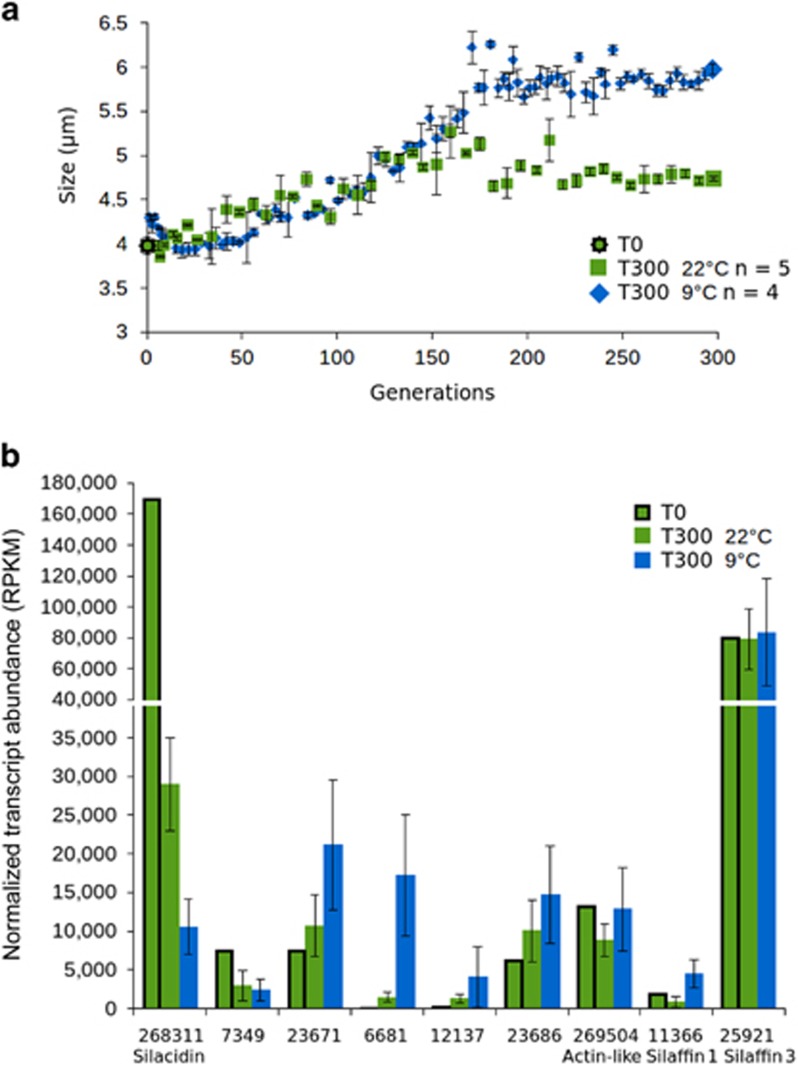
Cell size in *T. pseudonana* under experimental evolution conditions. (**a**) cell diameter measured as average particle diameter by coulter counter of cells grown for 300 generations at 22 °C (green) and 9 °C (blue). Error bars represent s.d. of measurements taken from biological triplicate samples. (**b**) Transcript abundance of silacidins; Protein IDs 7349, 23671, 6681, 12137 and 23686 found to be differentially requlated in TSD lines under late exponential and silicon-starved conditions and with expression patterns apparently similar to silacidins; cell wall genes silaffin 1 and silaffin 3; and an actin-like gene used as a housekeeping gene in transcriptomes of the same cell lines harvested after 300 generations at 22 °C (T300 22 °C, green), 9 °C (T300 9 °C, blue) and from batch culture nutrient conditions prior to the onset of the evolution experiment (T0 22 °C, green, with black outline). Error bars represent s.d. between transcriptomes sequenced from biological replicate samples (T300 22 °C *n*=5, T30 9 °C *n*=4).

*T. pseudonana* cell size was shown to respond to transfer from batch to semi-continuous culturing under experimental evolution, particularly at low temperature (9 °C). The average valve diameter expanded from 3.66±0.73 μm at T0 to, 4.97±0.73 μm at 9 °C and 4.19±0.55 μm at 22 °C after 300 generations (as measured by light microscopy and corresponding to coulter counter measurements of 3.98 μm, 5.93 μm and 4.73 μm, respectively, as shown in Figure 4). Silacidin transcript counts were extracted from transcriptome sequencing of the same samples (Schmidt *et al.*, in prep) and showed reduced silacidin transcripts concurrent with the increased cell size, whereas other genes encoding cell-wall proteins (silaffins 1 and 3) and a housekeeping actin gene did not have significantly different expression between the same samples (Figure 4). Silacidin expression decreased by 13.1±1.8-fold at 9 °C and 4.1±0.5-fold at 22 °C compared with T0 expression in batch culture. In addition, the transcript abundance for the 18 genes found be differentially regulated between both TSD lines and NAT under both late exponential and silicon-starved conditions were extracted from the transcriptome sequencing data from the experimental evolution experiment. One of these, ProteinID 7349 showed similar expression to that of silacidins, with lowest expression after 300 generations at 9 °C and highest expression under batch cultivation (T0). Four more, IDs 23671, 6681, 12137 and 23686, appeared to have opposite expression patterns with their lowest expression under batch cultivation (T0), and highest expression after 300 generations at 9 °C (Figure 4). The other 13 genes identified did not have regulation patterns in common with silacidin expression. Finally, RNA concentrations of cells in batch culture rose from 202.8 fg per cell to 293±74 fg per cell at 22 °C and 611±138 fg per cell at 9 °C after 300 generations under conditions of experimental evolution.

**Figure 5:**
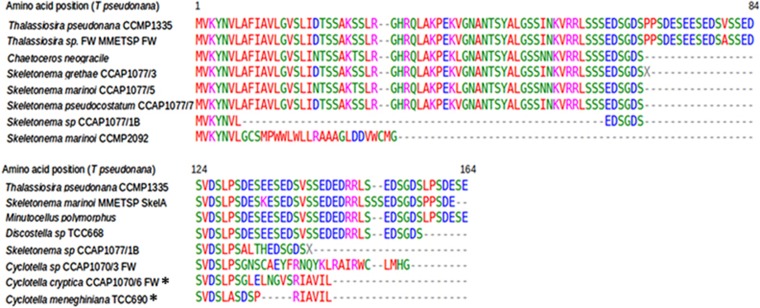
Identification of silacidin homologues in centric diatom species. Alignment of translated sequences from the species investigated. MMETSP denotes sequences obtained from the MMETSP transcriptome sequencing data set. Asterisks indicate sequences in which frameshift mutations were identified. Freshwater species are denoted ‘FW’.

To determine whether genes encoding for silacidins are present in other diatom species, we first examined available diatom genomes and found there were no homologues in the *Phaeodactylum tricornutum* or *Fragilariopsis cylindrus* genomes. We then examined the Marine Microbial Eukaryote Transcriptome Sequencing Project (MMETSP) data set for the silacidin gene sequence (Keeling *et al.*, 2014). Homologues were found in two centric diatom transcriptomes, a freshwater *Thalassiosira* species (MMETSP1059), and the estuarine species *Skeletonema marinoi* (MMETSP0920). These silacidin gene sequences had ⩾98% identity to the gene in *T. pseudonana*. All three gene sequences were used to design primers for a PCR approach to determine the presence of homologues in other centric diatom species. Eighteen cultures, comprising sixteen different centric diatom species were used, and amplification products were obtained and sequenced from 12 of them (Figure 5). It was difficult to amplify the gene encoding the silacidin protein owing to its highly repetitive nature. Only short sequences were generated (39–354 nt). Frameshift mutations were identified in two freshwater *Cyclotella* species (TCC690 and CCAP1070/6). In other species, translated sequences of the silacidin gene had 80–100% identity to the translated homolog in *T. pseudonana* (Figure 5).

## Discussion

Gene silencing has been a useful tool for elucidating unknown roles for specific diatom genes (for example, De Riso *et al.*, 2009; Lavaud *et al.*, 2012; Levitan *et al.*, 2015 and Shrestha and Hildebrand, 2015). However, the molecular mechanism underlying gene expression deregulation is still unknown. Here, we used the gene silencing approach to deregulated silacidine gene expression. Gene silencing was not apparent at the transcript level as the silacidin transcript abundance of transgenic lines containing gene-silencing vectors was not significantly different to wild-type lines according to qPCR and transcriptome sequencing. However, reduced silacidin protein abundance was observed in biosilica in transcending lines containing constructs for TSD lines compared with NAT control cell lines according to size exclusion chromatography. This suggests that deregulation of silacidin expression likely occurs by preventing translation of the mRNA rather than by triggering degradation of the mRNA transcripts. Similarly, gene deregulation has been reported to give clear phenotypes in the absence of reduced transcripts in *P. tricornutum* (De Riso *et al.*, 2009, Lavaud *et al.*, 2012), and *T. pseudonana* (Shrestha and Hildebrand, 2015), and transcript abundance appears to vary depending on the gene targeted for deregulation. These observations are true whether antisense of inverted repeat constructs are used (for example, Molnar *et al.*, 2010). Finally, even if the TSD lines do not allow to establish a clear molecular link between deregulation of silacidin expression and cell size reduction, they still have revealed a genetic control of cell size in diatoms.

The increased valve diameter associated with deregulation of silacidin gene expression and differential regulation of 18 genes (13 upregulated and 4 downregulated, and 1 upregulated in late exponential phase and downregulated under silicon starvation in TSD compared with control cell lines) in *T. pseudonana* implies that silacidins may be part of a wider network of genes restricting the size of valves and therefore the cell size of centric diatoms. In most diatom species, cell diameter decreases over generations as new hypotheca are synthesized within the parent cell restricted by the diameter of the mature valves (Macdonald, 1869). When a critical diameter is reached, auxosporulation and sexual reproduction is initiated restoring the maximum cell size (Crawford, 1974). However, *T. pseudonana* has been shown not to undergo successive cell-size decrease and rarely undergoes auxosporulation (Hildebrand *et al.*, 2006) but other centic diatoms, which have the silacidin gene encoded (for example, *C. meneghiniana, S. marinoi*), have well documented sexual cycles including successive cell-size decrease (Schultz and Trainor, 1970; Godhe *et al.*, 2014). This may indicate that silacidins and associated regulators (for example, ProteinID 264048: protein kinase with proline-rich region) are possibly responsible for successive cell-size reduction during sexual cycles in centric diatoms. For species that do not undergo frequent sex (for example, *T. pseudonana*), those proteins may be used to stay small especially under stressful conditions such as nutrient limitations where a large surface-to-volume ratio is beneficial for nutrient acquisition. The silacidin protein is highly abundant especially under silicate limited growth (Richthammer *et al.*, 2011; Figure 1), which corroborates this assumption.

Cell-size plasticity is also required to respond to changing environmental conditions that take place on timescales relevant for evolutionary adaptation. The diatom fossil record demonstrates that small diatoms dominated in warm conditions when increased surface area to volume ratios increases drag and slows sinking speed maximising residency time in the photic zone, and increase nutrient uptake efficiency at low concentrations. Thus, cell size responds to environmental pressure over evolutionary time, by selection for species of larger or smaller size according to turbulence and temperature trends (Falkowski and Oliver, 2007). Here, we observed a comparable size-response to low temperatures and constant sufficient availability of nutrients over simulated evolutionary timescales (Figure 4). *T. pseudonana* cell diameter increased in response to decreased temperatures and semi-continuous growth that provided uninterrupted supply of nutrients similar to diatom growth in coastal upwelling systems. At both temperatures, cultures went from batch with interrupted supply of nutrients to semi-continuous culturing. These experiments give evidence that both, low temperature and sufficient nutrient availability individually increase the cell size of *T. pseudonana*, which is accompanied by strong downregulation of the gene encoding the silacidin protein and associated genes which we identified in the transcriptomes. Other genes encoding cell-wall proteins (silaffins) and control genes did not change their expression in the evolved cells, strengthening the role of silacidins for cell-size plasticity in *T. pseudonana*.

Size is an important aspect of the cellular shape and it affects internal cellular architecture. Both are controlled by the coupling of cell growth (for example, production of girdle bands in diatoms) and division. Perturbation of silacidin gene expression, though a still unknown mechanism, has allowed us to obtain new insights into how cell size in diatoms affects key physiological processes such as cell division (growth rates), photosynthesis, nucleic acid content and nutrient uptake. Most of the earlier studies addressing the role of cell size for these physiological processes were based on interspecies comparisons, therefore potentially including species-specific differences unrelated to differences in cell size. Our study showed that growth rates were not affected by cell size, although the generation of larger cells may be expected to be energetically more expensive. Growth efficiency has been shown not to be size-dependent in various unicellular algae (Banse, 1976), and within the diatom *Ditylum brightwellii*, a centric diatom whose cell size decreases according to the McDonald-Pfitzer hypothesis during vegetative growth, the division rates of significantly different sized cells vary little (Sharpe *et al.*, 2012). The quantum yield of photosynthesis (Fv/Fm) did not differ under exponential growth between smaller and larger cells of *T. pseuodnana* but became more pronounced under stationary growth (Figure 2) with larger cells having higher Fv/Fm. This is surprising as larger cells tend to reach stationary phase earlier with reduced cell densities (Figure 2bii), which should have increased the stress level owing to a longer exposure to nutrient limitation and higher irradiance due to lower cell numbers. Maybe the decreased surface-to-volume ratio of the larger cells reduced the absorbance of light and therefore light stress under nutrient limitation. The construction of larger cells (with larger frustules) also required more silicic acid (Figure 2aii) and nitrate per cell (Figure 2b), as observed by lower yields of larger cells in media limited by these components. Larger cells also contained more RNA per cell in both TSD and experimental evolution experiments.

Our comparative analysis including transcriptomes from both TSD cell lines was conducted to identify additional genes involved in cell-size modification of *T. pseudonana*, which has never been done before for any marine alga. This work led to the identification of 18 candidate genes of which most have no known function. However, the putative protein kinase with a proline-rich region (ProteinID 264048) is a promising candidate for controlling cell size in *T. pseudonana* as the main cell-size controller in yeast is also a kinase (Wee1) and the proline-rich region potentially indicates cell wall localisation. However, there is no sequence similarlity between Wee1 from yeast and the *T. pseuodnana* protein (ProteinID 264048) (data not shown). Genes most likely involved in secondary effects of cell-size enlargement were encoding proteins with possible roles in cell adhesion, which was reduced in larger cells, hence these genes were downregulated (for example, cysteine-rich secretory protein, Fibronectin type III). Under late exponential growth, chitinases and chitin-binding interpro domains were overrepresented in both TSD cell lines. Chitin may be in the form of extruded chitin fibres thought to influence sinking behaviour in diatoms, or in the form of a mesh-like component of the cell wall (Brunner *et al.*, 2009). The larger frustule, but smaller surface area to volume ratios of the TSD cells may influence the expression of chitin metabolism genes compared with the rest of the transcriptome.

Despite the identification of the silacidin gene and additional genes potentially involved in cell-size plasticity in *T. pseudonana,* our set of genes by all means is not comprehensive and not causative enough to elucidate the complex regulatory mechanisms of cell-size modification in centric diatoms. However, it is the first study for any marine alga that relates individual genes with changes in cell size and our data give first evidence that the cell wall has a significant role potentially in accordance with regulation of the cell cycle as both processes are tightly coupled in diatoms. In yeast, addition of new membrane to the cell surface by membrane trafficking is necessary for cell growth (Anastassia *et al.*, 2012). The delivery of vesicles to the cell surface generates a signal that is proportional to cell growth and therefore is integrated with cell-cycle progression and potentially also cell size and morphogenesis. Some of the 18 genes identified in our transcriptome study encode proteins known to be involved in vesicle transport (for example, synaptobrevin) and harnessing the energy from a proton gradient across membranes (for example, V-ATPases), suggesting the presence of similar processes in *T. pseudonana.*

Secondary effects not directly related to the modification of the cell size were observed for both phenotypes of TSD cell lines and their transcriptomes such as reduced aggregation of the enlarged cells accompanied by downregulation of genes encoding for proteins involved in adhesion (Fibronectin). These secondary effects blur the causative nature between deregulation of the silicidin gene (cause) and the increase in cell size (effect). Furthermore, the process of gene deregulation and the lack of knowledge about its mechanism in diatoms might have had an impact on our results as we saw many genes differentially regulated that were not in common between both TSD cell lines. However, an increase in cell size was still observed in both independently generated TSD cell lines. The effect of cell-size enlargement could have also been impacted by processes we did not address in our study such as changes in the ribosome content or the redundancy of processes underpinning silica deposition and cell-wall formation in diatoms. For instance, in yeast, the lack of components of ribosomes causes the formation of significantly smaller cells (Soifer and Barkai, 2014). A recent study with diatoms including *T. pseudonana* showed that low temperatures (4 °C) increase cellular concentrations of ribosomes (Toseland *et al.*, 2013), which could have contributed to increasing cell size in our evolution experiments at 9 °C. Silacidins belong to a larger group of proteins able to precipitate silica for cell-wall formation. How this redundancy could have impacted the phenotype we observed in our study remains elusive but should be addressed in subsequent studies. Using the latest reverse genetics tools including genome editing (CRISPR-Cas, Hopes *et al.*, 2016) in combination with biochemical studies most likely will pave the way for new insights into processes regulating the cell size in diatoms, which after all underpins food-web structure in the marine system and global biogeochemical cycles.

## Figures and Tables

**Table 1:** Number of differentially regulated genes (⩾2-fold (1 × log^2^), *P*-value<0.01) between transgenic lines with targeted silacidin deregulation (TSD) and NAT cultures in common with genes identified in previous studies (Shrestha *et al.*, 2012; Mock *et al.*, 2008)

*Conditions*	*TSD1 E*	*TSD3 E*	*TSD1 S*	*TSD3S*	*TSD1+3 E*	*TSD1+3 S*	*TSD1+3 E+S*	*Total*
SLRG	51	36	54	24	7	15	1^a^	485
SSRG	29	8	63	21	4	6	2^b^	534
Mock_Si	142	101	164	108	13	45	2^c^	822
Mock_Fe	121	45	92	59	0	1	0	466
Mock_N	57	39	76	57	2	18	0	627
Mock_T	85	55	139	109	1	13	0	989
Mock_pH	55	42	64	57	6	25	2^d^	377
None of above	423	369	731	465	53	153	12^e^	
Total	702	553	1126	707	77	236	18 (see note)	

Abbreviations: E, late exponential phase; S, stationary phase; TSD1, transgenic targeted silacidin deregulation line 1; TSD3, transgenic targeted silacidin deregulation line 3; SLRG, Silaffin*-*like response genes; SSRG, silicon-starvation responsive genes, (Shrestha *et al.*, 2012); Mock _Si (silicate limitation), _Fe (iron limitation), _N (nitrate limitation), _T (low temperature), _pH (elevated pH), (Mock *et al.,* 2008). Note: Gene ID 23685 features in both Mock_Si and Mock_pH.

Genes differentially regulated in all TSD transcriptomes compared with NAT (TSD1+3 *E+S*).

a Gene ID 264048 (GO terms 0004672 protein kinase activity, 0004674 protein serine/theonine kinase activity, 0005199 structural component of cell wall, 0005524 ATP-binding, 0006468 protein amino acid phosphorylation).

b Gene ID 8776 (no annotation), Gene ID 9371 (no annotation).

c Gene ID 23685 (no annotation), Gene ID 23671 (no annotation).

d Gene ID 23685 (no annotation), Gene ID 7349 (no annotation).

e Gene IDs 8615, 3898, 8616, 6681, 7687, 12137 (no annotation), Gene ID 7353 (IPR000408 Regulator of chromosome condensation, RCC1), Gene ID 6886 (IPR001440 TPR repeat), 7435 (GO terms 0016051: carbohydrate biosynthetic process, 0016758 transferase activity transferring hexosyl groups), 263350 (GO terms 0003899 DNA- directed RNA polymerase activity, 0006350 transcription) 23686 (IPR001678 Bacterial Fmu (Sun)/eukaryotic nucleolar NOL1/Nop2p), 9840 (GO terms 0003677 DNA binding, 0003700 sequence-specific transcription factor activity, 0005215 transporter activity, 0005634 nucleus, 0006355 regulation of transcription DNA-dependent, 0006810 transport, 0016020 membrane).
