# Supplementary material for: A role for the cell-wall protein silacidin in cell size of the diatom Thalassiosira pseudonana
Source: ISME J. 2017 Jul 21;11(11):2452–64. doi: 10.1038/ismej.2017.100 (PMC5649158; doi:10.1038/ismej.2017.100)
Supplement: Supplementary Figure S1 [file ismej2017100x7.ppt]

## Slide 1
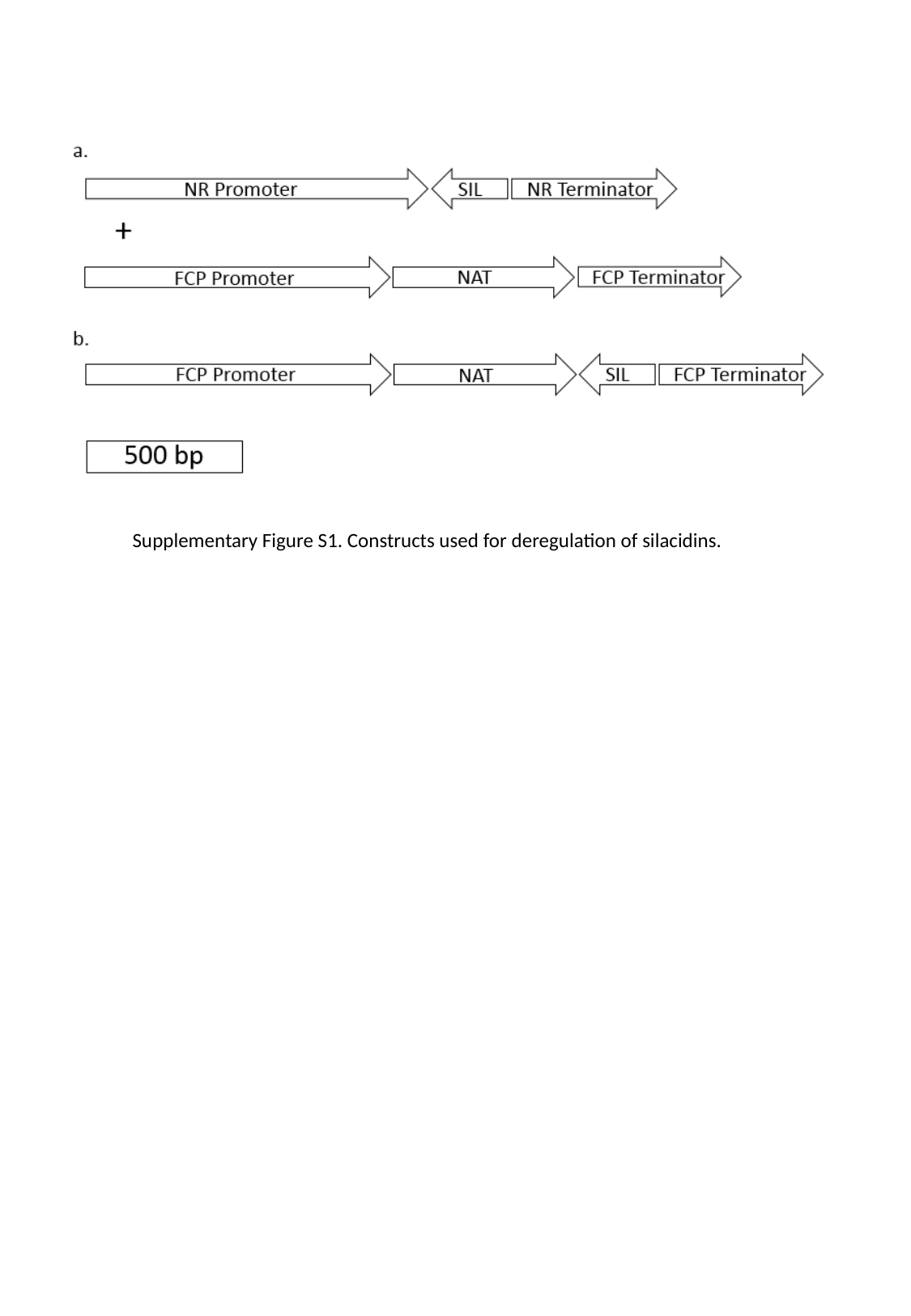

Supplementary Figure S1. Constructs used for deregulation of silacidins.

## Slide 2
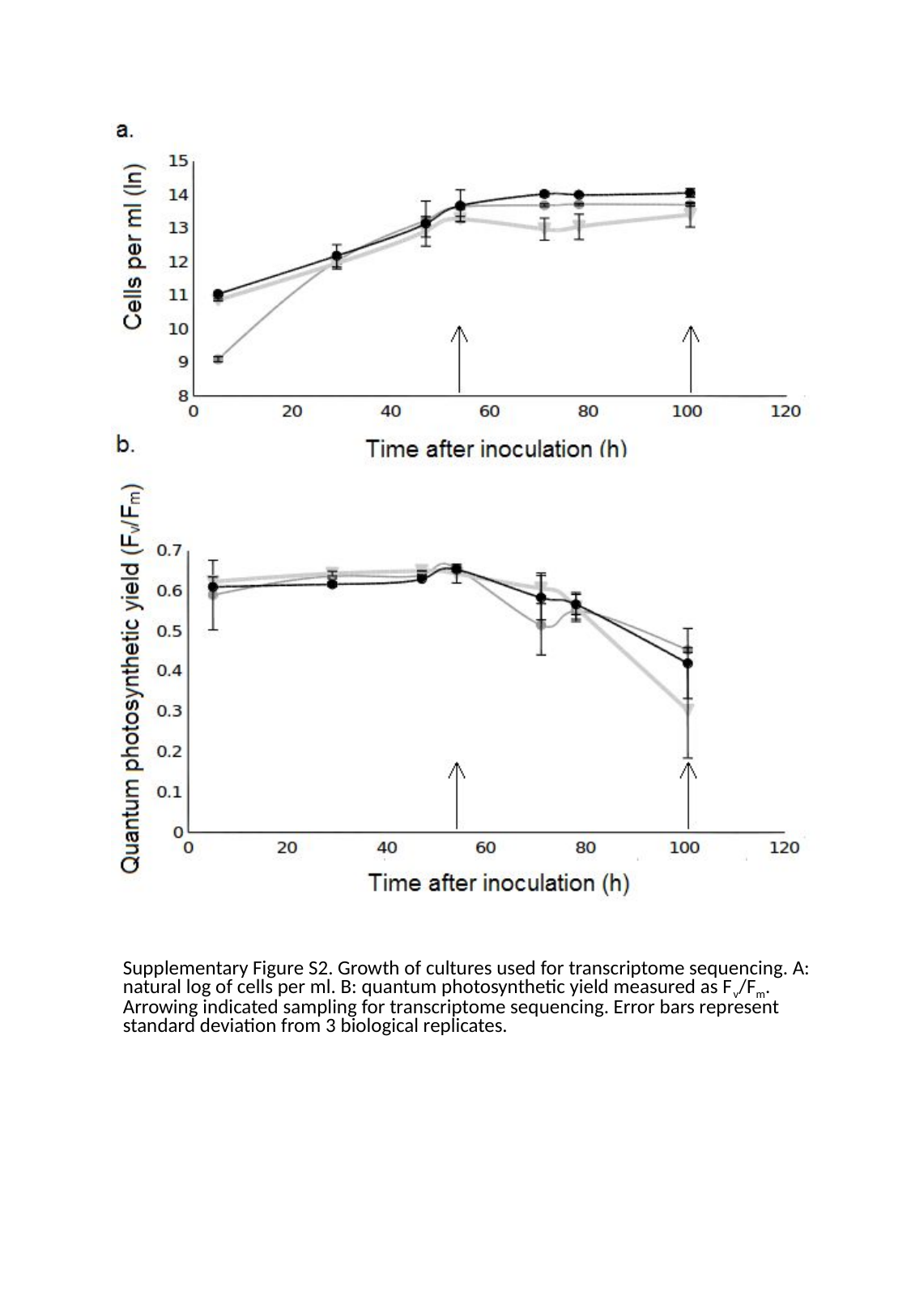

Supplementary Figure S2. Growth of cultures used for transcriptome sequencing. A: natural log of cells per ml. B: quantum photosynthetic yield measured as Fv/Fm. Arrowing indicated sampling for transcriptome sequencing. Error bars represent standard deviation from 3 biological replicates.

## Slide 3
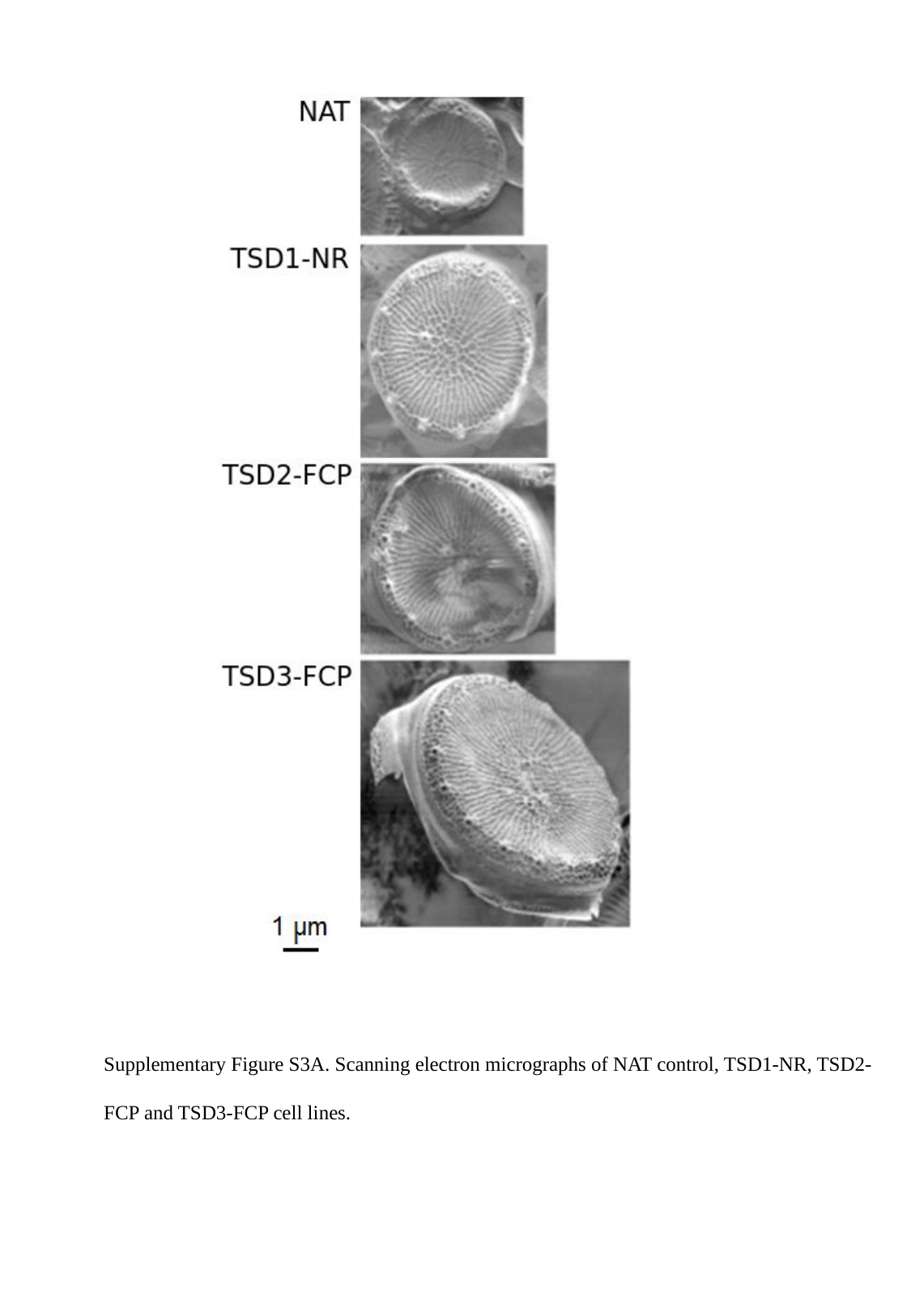

Supplementary Figure S3A. Scanning electron micrographs of NAT control, TSD1-NR, TSD2-FCP and TSD3-FCP cell lines.

## Slide 4
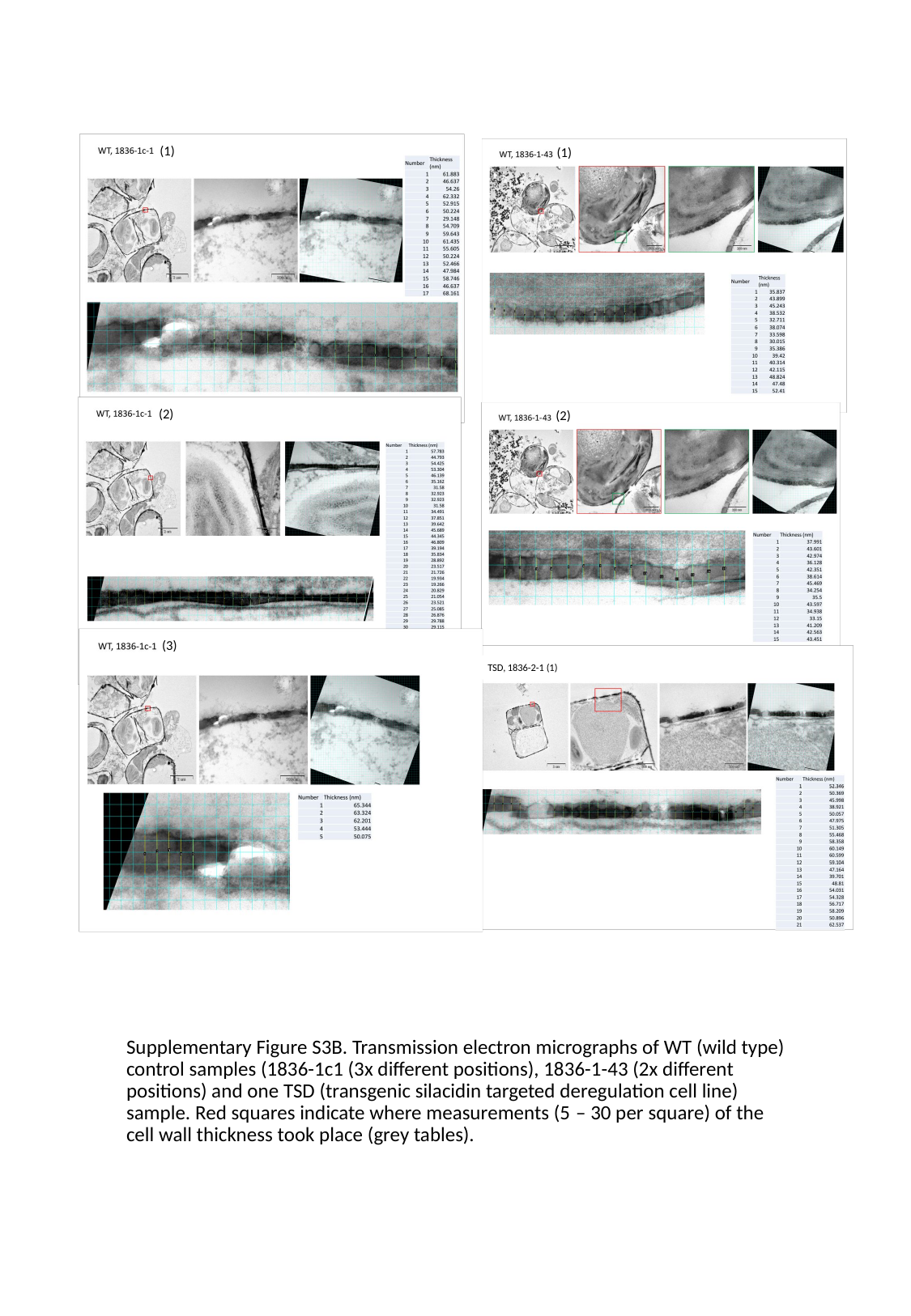

(1)
(1)
(2)
(2)
(3)
(1)
TSD, 1836-2-1 (1)
Supplementary Figure S3B. Transmission electron micrographs of WT (wild type) control samples (1836-1c1 (3x different positions), 1836-1-43 (2x different positions) and one TSD (transgenic silacidin targeted deregulation cell line) sample. Red squares indicate where measurements (5 – 30 per square) of the cell wall thickness took place (grey tables).

## Slide 5
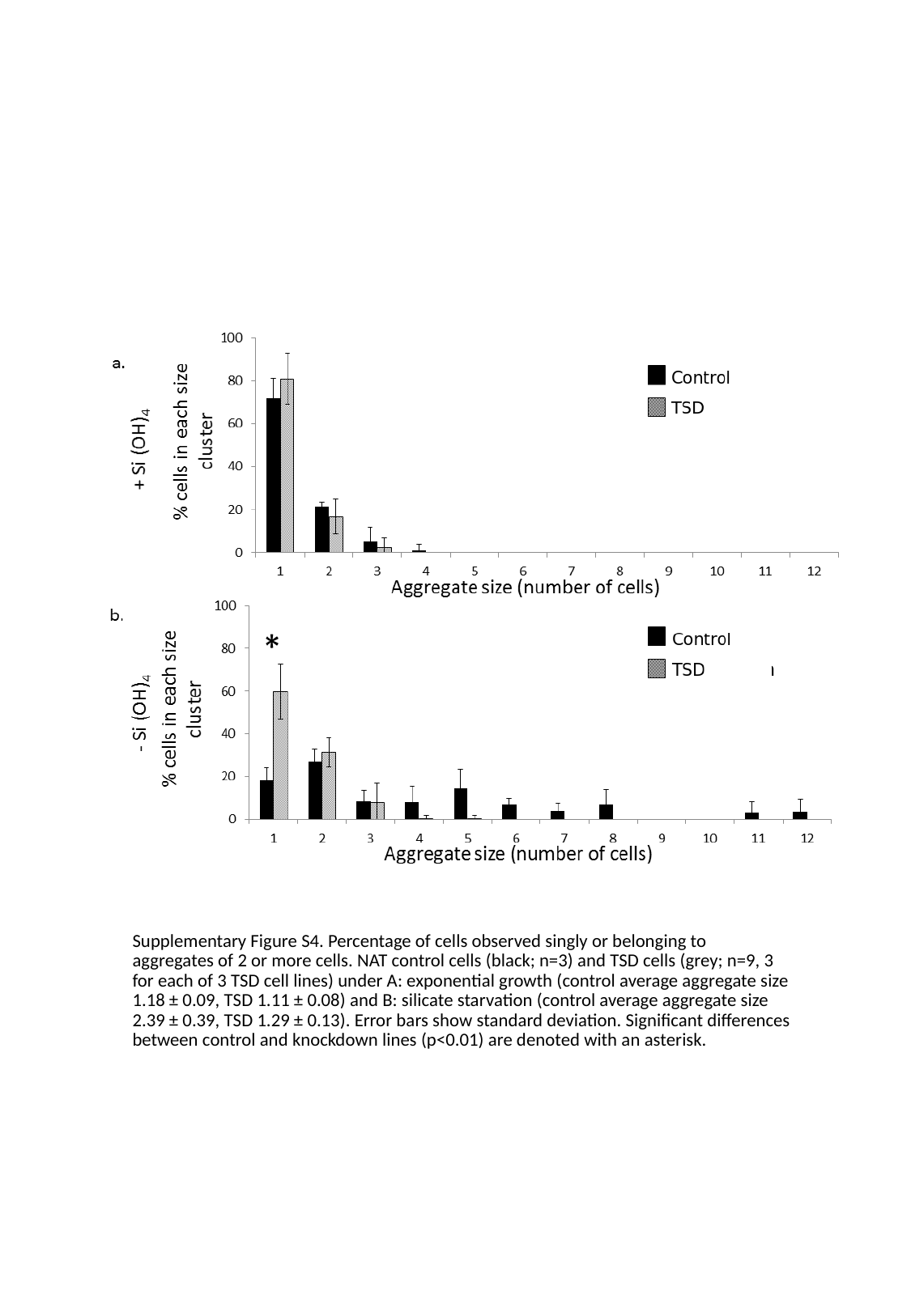

Supplementary Figure S4. Percentage of cells observed singly or belonging to aggregates of 2 or more cells. NAT control cells (black; n=3) and TSD cells (grey; n=9, 3 for each of 3 TSD cell lines) under A: exponential growth (control average aggregate size 1.18 ± 0.09, TSD 1.11 ± 0.08) and B: silicate starvation (control average aggregate size 2.39 ± 0.39, TSD 1.29 ± 0.13). Error bars show standard deviation. Significant differences between control and knockdown lines (p<0.01) are denoted with an asterisk.
